# Recent Trends in Nanofibrous Membranes and Their Suitability for Air and Water Filtrations

**DOI:** 10.3390/membranes1030232

**Published:** 2011-08-22

**Authors:** Ramalingam Balamurugan, Subramanian Sundarrajan, Seeram Ramakrishna

**Affiliations:** 1 NUSNNI, National University of Singapore, 2 Engineering Drive 3, Singapore 119077, Singapore; 2 Department of Mechanical Engineering, National University of Singapore, 2 Engineering Drive 3, Singapore 119077, Singapore; 3 Institute of Materials Research and Engineering, Singapore 117602, Singapore; 4 King Saud University, Riyadh 11451, Kingdom of Saudi Arabia

**Keywords:** electrospinning, air filter media, water treatment membranes, textiles, chemical and biological contaminants, nanoparticles, micro-, ultra-, nanofiltrations

## Abstract

In recent decades, engineered membranes have become a viable separation technology for a wide range of applications in environmental, food and biomedical fields. Membranes are now competitive compared to conventional techniques such as adsorption, ion exchangers and sand filters. The main advantage of membrane technology is the fact that it works without the addition of any chemicals, with relatively high efficiency and low energy consumption with well arranged process conductions. Hence they are widely utilized in biotechnology, food and drink manufacturing, air filtration and medical uses such as dialysis for kidney failure patients. Membranes from nanofibrous materials possess high surface area to volume ratio, fine tunable pore sizes and their ease of preparation prompted both industry and academic researchers to study their use in many applications. In this paper, modern concepts and current research progress on various nanofibrous membranes, such as water and air filtration media, are presented.

## 1. Introduction

Chemical and biological contaminants present in environments such as in air and water sources are a constant concern for human health. For the betterment of human life, before consumption of air and water, these pollutants are to be eliminated from their sources. Conventionally various materials (such as activated carbon) and various methods (sediment deposition and adsorption and many more), were utilized to remove pollutants. Although some of them were efficient and convenient, they have some drawbacks. In order to develop new and innovative materials, several researchers have explored various materials for such applications.

**Figure 1:**
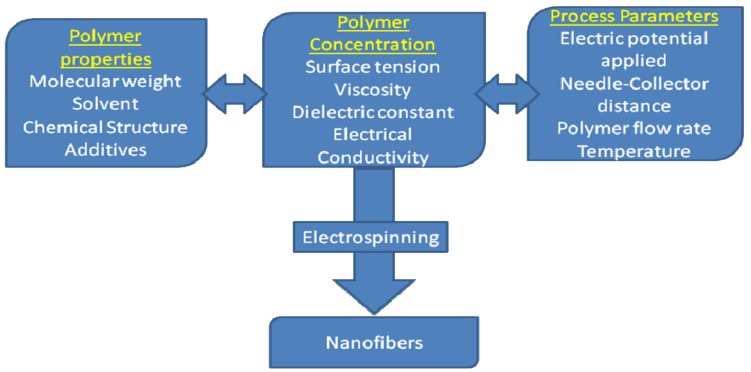
Overall parameters involved in electrospinning methodology.

Among them, nanofibers are one of the most important nanostructured materials studied for various applications such as healthcare, energy, catalysis, electronics, protective clothing, bioengineering and biotechnology and environmental applications. The major parameters governing electrospinning technology are given in Figure 1. Electrospun nanofibers possess high density of pores, high surface area to volume ratio, high permeability, low basis weight and small fiber diameter [1]. These properties are suitable to be used as filtering media in water and air filtration applications. Although, polymeric nanofibers are currently employed for commercial applications in air filtrations, they are yet to be exploited for real applications in water domain. Also, the tendency of the nanofiber membranes to selectively permeate moisture and thereby enhance breathability and block the chemical vapors made them suitable to be applied in air filtration applications such as in protective clothing domain in textile industry [2] for protection against chemical and biological (CB) warfare contaminants [3,4,5,6,7].

In the case of water filtration applications, depending on the pore size and filtration application, the purification process can be categorized as microfiltration (MF), ultrafiltration (UF), nanofiltration (NF), reverse osmosis (RO), and forward osmosis (FO). Among them, nanofibrous membranes were studied for MF and UF application because of their ability to reduce the resistance to water flow (*i.e.*, high water flux) [8,9,10] and NF application for providing water channels in the barrier layer thereby minimizing fouling [11,12]. Nanofibers were also employed as affinity membrane for both biological [13,14,15] and wastewater treatment applications [16]. The filtering media prepared from nanofibers possess enhanced performance in air filtration application. This performance has the potential in future to revolutionize the current water filtration technology by providing cheaper and portable units consuming less energy.

In this paper, current research on progress of various nanofibrous membranes made in water and air filtration media and future directions are presented. One of the drawbacks of the electrospun nanofibers for use in air and liquid filtration applications is that they are mechanically unstable when compared to cast membranes made from the same polymer. In this article, recent developments in this domain and the ability to enhance the filtration performance in presence of support material are also highlighted.

## 2. Results and Discussion

### 2.1. Air Filtration Applications

Currently charcoal and glass fibers are the two most important materials widely used in air filtration applications. Their usages in different areas of applications are summarized in the forthcoming sections separately.

#### 2.1.1. Protective Clothing Applications

##### 2.1.1.1. Conventional Materials

Conventionally, charcoal impregnated with metal oxides such as Ag, Cu, Zn, and Mo in the presence of triethylene diamine (TEDA) has been used in the existing protective clothing (and in face masks) applications [16]. Two types of protective suits, *i.e.* impermeable and permeable protective suits, were widely used by soldiers to protect against chemical and biological contaminants. Low vapor pressure (below 10 mmHg) contaminants (such as nerve and vesicant) were removed by physical adsorption (through pores), whereas high vapor pressure contaminants (blood and choking agents) were removed by chemical reaction. Some drawbacks of the existing protective clothing are: heavy, moisture adsorption, and disposal after use. Hence, in order to overcome the drawbacks in the existing technology, various studies are currently in progress in several laboratories (especially in US army) and in our lab at NUS. Some of the recent developments made in our lab at NUS in this area are highlighted below.

##### 2.1.1.2. Recent Trends—Combination of Nanoparticles with Nanofibers

**Figure 2:**
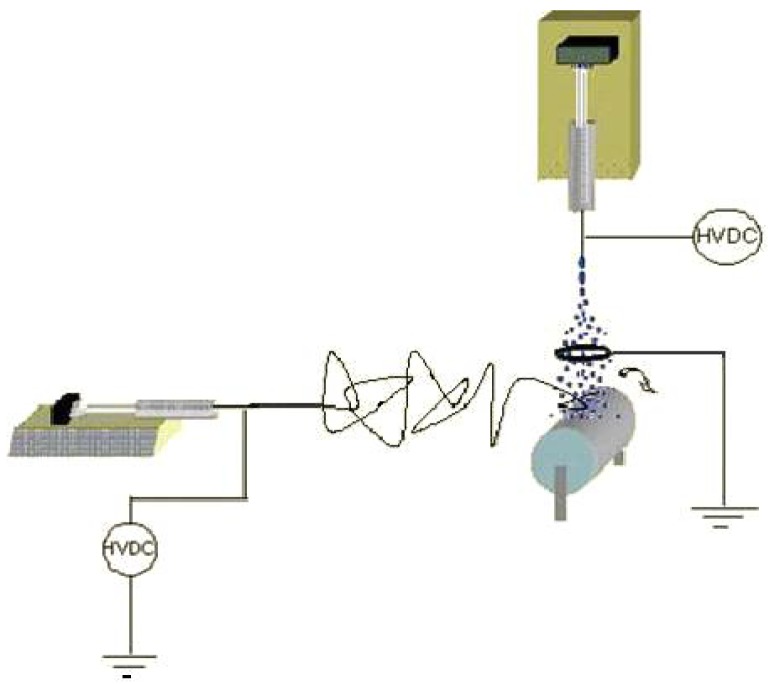
Schematics of the experimental set up used for the combination of electrospinning and electrospraying techniques (Adapted from [18]).

The incorporation of nanoparticles such as MgO, TiO_2_, Al_2_O_3_ and other oxides into nanofibers was recently explored by some of our researchers for air filtration applications. This is because of the unique ability of nanoparticles to decontaminate wide varieties of toxic gases, such as chemical contaminants, biological contaminants (viruses, bacteria), pesticides, and many more. Recently, the nanoparticles were incorporated into nanofibers by using various methods in our lab and protective clothing applications were studied [3,6,17]. After mixing the nanoparticles with polymer solutions followed by electrospinning, the obtained nanocomposite membranes were tested for the decontamination of the stimulant of nerve gas, paraoxon. However, the nanoparticles were covered by polymer material and were therefore not fully available on the surface for catalytic applications [3]. To overcome this problem, electrospraying was combined with the electrospinning technique for the first time in the literature and the typical experimental set up used is shown in Figure 2.

**Figure 3:**
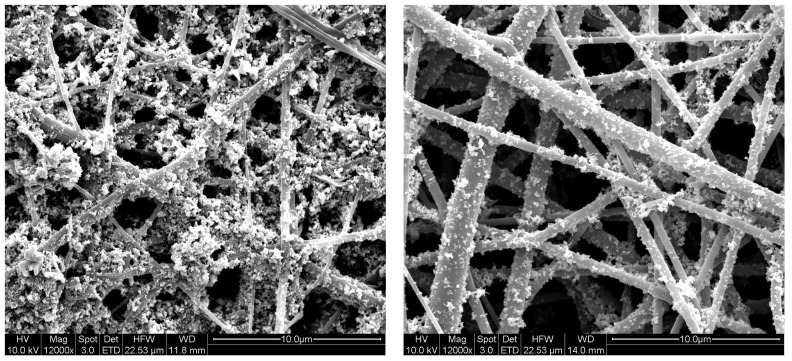
SEM images of dip coated samples of CA/PET-ZnO (left) and PET-ZnO (right) (Adapted from [18]).

Electrospinning of nanofibers and electrospraying of nanoparticles were carried out either simultaneously or one by one and we have demonstrated that this technique is an efficient and viable method for the preparation of nanocomposite membranes for air filter applications [6]. In addition, it has been proved that pressure drop decrease was dependent upon the amount of the nanoparticles electrosprayed. One of the drawbacks of nanoparticles based nanofibrous filters is that the stability of nanoparticles over nanofiber may be poor. Recently, Sundarrajan *et al.* applied a concept of functional group containing polymers, wherein the nanoparticles can bind with the functional groups (ethylene imine) present on the surface [18] and stability was compared with non functional group containing nanofiber, such as nylon by methanol washing. It has been observed that more particles were washed away from nylon surfaces. When dip coating technique was applied to coat the nanoparticles, more particles were nucleated on the functional group containing nanofiber surfaces such as on (poly(ethylene terephthalate) (PET) and cellulose or PET and cellulose acetate (CA) when compared to the PET surfaces (Figure 3). This is because of the presence of hydroxyl functional groups on the cellulose or cellulose acetate surfaces [18].

We believe that with decreased flow resistance and improved performance, these membranes can be applied as air-con filters (in hospitals, airplanes, industry and many more), protective clothing, textile and other applications. The exploration of these membranes for air filter application is in progress in our laboratory.

#### 2.1.2. Textile Applications

Textiles is one of the industries, wherein nanotechnology products are commercially applied to protect humans and their environment. The nanomaterials are embedded into textile products to impart antimicrobial properties, decrease luster, and protect against UV rays. When compared to conventional materials, nanomaterials offer several advantages such as needing lesser amounts of nanomaterials and enhancing product's performance. To cite a few: metal oxide nanoparticles to decrease luster or provide UV protection [19]. Alkoxysilane-modified TiO_2_ nanoparticles to absorb UV radiation [20], and Ag, TiO_2_, and ZnO nanoparticles to provide antimicrobial and UV protection properties [7,21] and monazite as thermal protection blankets for reentry space craft application [22].

#### 2.1.3. Clean Air Applications in Hospitals and Other Domains

In the previous sections, the exploration of nanofibers for various applications was summarized. We believe that the potential application of nanofibers is enormous and one of the breakthrough domains in future will be to use them as filter media in clean air applications in hospitals. This idea is based on the fact that Ahn *et al.* have studied the filtration efficiency of nylon-6 nanofibrous membranes, which is better than the commercialized high-efficiency particulate air filter (HEPA). One of the drawbacks is that they observed high pressure drop across the membrane [23]. However, this study suggests that they can be potentially employed as HEPA filter with high efficiency in clean air applications such as in hospitals (and other applications) wherein the contaminated air (bacteria and other pathogens) in a room can be filtered before entering into other rooms due to centralized air conditioning systems. Recently, nanofibers were explored for clean air applications by various companies. For example, United Air Specialists, Inc., has highlighted some of the salient features of nanofibers in their company website that they provide high efficiency, less energy cost, longer filter life, and greater value due to lower cost per cubic feet per minute (CFM) [24].

#### 2.1.4. Mechanical Properties of Nanofibers

Generally, the mechanical properties of polymer nanofibers are worse when compared to textile fibers and film made from the same polymer. This is not only because the polymer molecules are not aligned fully during stretching at the time of electrospinning, but also due to reduced interaction between polymer molecules in nanofibers. Recently, it has been reported that the mechanical properties of nanofibers are directly proportional to the fiber diameter. However, Huang *et al.* [25], reported a tensile strength of 664 MPa and a tensile modulus of 15.3 GPa for a rigid-rod-like macromolecule such as polyimide polymer (poly(p-phenylene biphenyltetracarboximide)) by aligning the PI nanofibers. These nanofibers with excellent mechanical properties can be applied to protective clothing, water and other applications. Recently, nanoparticles were electrosprayed over nanofibers by Velmurugan *et al.* [26]. They showed that the addition of a smaller amount of TiO_2_ nanoparticles increases the mechanical properties (0.36 MPa for polyimide and 0.65 MPa for polyimide/TiO_2_ membrane) of nanofibrous membrane.

#### 2.1.5. Commercial Applications

Exploitation of nanofibers has been pursued by various companies (Donaldson, Eden Energy Limited) for various commercial applications (for example, air, battery). Some of the commercial suppliers of nanofibers for environmental applications are given in Table 1.

**Table 1:** Some of the Commercial suppliers of Nanofibers for Environmental Applications.

**S. No.**	**Companies**	**Country of origin**	**Website** *
1	Donaldson Company Inc.	USA	www.donaldson.com [27]
2	Espin Technologies Inc.	USA	www.espintechnologies.com [28]
3	KX Industries	USA	www.kxindustries.com [29]
4	Ahlstrom Corporation	Finland	www.ahlstrom.com [30]
5	Hollingsworth Co. Ltd.	USA	www.hollingsworth-vose.com [31]
6	US Global Nanospace	USA	www.usgn.com [32]
7	Finetex Technology	S. Korea	www.finetextech.com [33]
8	Helsa-automotive	Germany	www.helsa-automotive.com [34]
9	Teijin Fibers Ltd.	Japan	www.teijinfiber.com [35]
10	Toray	Japan	www.toray.com [36]
11	Japan Vilene Company Ltd.	Japan	www.vilene.co.jp [37]
12	Nanoval GmbH & Co. KG	Germany	www.nanoval.de [38]
13	Hills Inc.	USA	www.hillsinc.net [39]
14	Elmarco	Czech Republic	www.elmarco.cz [40]
15	Hohns Manville Sales GmbH	Germany	www.jmeurope.com [41]
16	Esfil Tehno	Republic of Estonia	www.esfiltehno.ee [42]
17	Sorbent	Russian Federation	www.sorbent.su [43]
18	Electrostal Chemico-Mechanical Factory	Russian Federation	www.ehmz.ru [44]
19	Neorganika	Russian Federation	www.neorganika.ru [45]
20	Kimry's factory	Russian Federation	www.fgsiz.ru [46]
21	A.A.Gunyaev NW R&D Center “Lightweight PPE”	Russian Federation	www.psiz.ru [47]
22	Progress-Ecologia	Russian Federation	www.p-ecologia.obninsk.ru [48]
23	“NPP” Doza	Russian Federation	www.doza.ru [49]
24	Engineering Research centre “SNIIP”	Russian Federation	www.sniip.ru [50]

* Websites were accessed on 10 August 2011.

#### 2.1.6. Nanofibrous Membranes in Environmental Applications

Today, an electrospun nanofiber with engineered nonwovens produces a variety of new materials previously utilized in daily basic human needs like air, water and bio-pharmaceutical related products. The nonwoven webs of fibers produced from the electrospinning process have high specific surface areas, nano scale pore sizes, high and controllable porosity and extreme flexibility with regard to the materials used and modification of the surface chemistry of the fibers. Ramakrishna and co-workers extensively reviewed the development and engineered nanofibrous membranes for environmental applications [51]. Nanofiber membrane separation process can be introduced into numerous industrial applications due to their advantages like appreciable energy savings, environmentally benign, clean technology with operational ease, replacing conventional processes, and producing high quality products, with greater flexibility in designing systems. The tremendous improvement in nanofiber production technology has paved the way to use them in major liquid separation methods like micro (MF), Ultra (UF) and nanofiltration (NF). In this part of the review, we will discuss recent trends and the major roles of nanofibers in the water purification applications in MF, UF and NF domains.

#### 2.1.7. Types of Nanofibrous Membranes and Their Bio-Removal Applications

**Figure 4:**
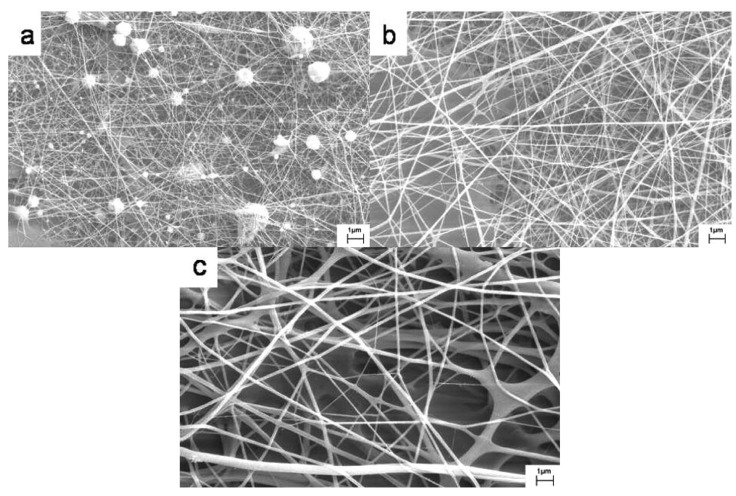
SEM images of HMW chitosan/HMW PEO blend fibers: **(a)** 1.33 wt% HMW chitosan/HMW PEO (90:10); **(b)** 1.6 wt% HMW chitosan/HMW PEO (75:25); **(c)** 2.00 wt% HMW chitosan/HMW PEO (50:50) (Adapted from [52]).

The important key role in electrospinning is the selection of polymers with suitable solvents. Nanofiber structure formed was characterized by fiber shape and size and was found to be strongly dependant on the polymer molecular weight, their blend ratios, polymer concentration, and choice of solvent. Many of the naturally occurring polymers cannot be electrospun due to their limited or poor solubility in organic solvents. However, they can be electrospun using toxic solvents such as trifluroaceticacid, which is harmful to the environment. Desai *et al.* successfully electrospun a natural polymer such as chitosan after overcoming the poor solubility in organic solvents by blending with low percentage of PEO using acetic acid as solvent [52] and they confirmed the formation of nanofibers by using SEM (Figure 4). They observed that uniform fiber pattern and size were mostly influenced by blend ratio of polymers, polymer concentration/weight and solvent. Further, they have proved the presence of both the polymers in the nanofibers by thermo gravimetric analysis (TG).

**Figure 5:**
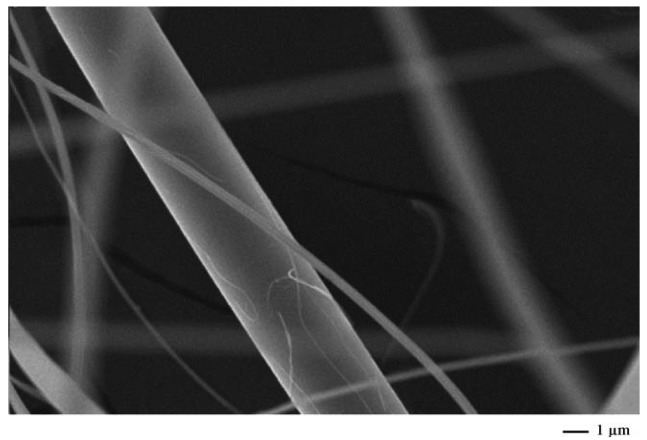
SEM picture of electrospun EPS nanofiber and commercial glass fiber (Adapted from [53]).

Shin *et al.* used the recycled expanded polystyrene nanofibers for filter media which used to replace the micro glass fibers. Micro glass fibers are mostly used in petrochemical industries for water in oil emulsion separation. Introduction of expanded polystyrene nanofibers to conventional nanofibers increases the separation efficiency of the filter media by 20% [53]. SEM picture reveals the mixed glass and electrospun expanded polystyrene nanofiber (Figure 5).

**Figure 6:**
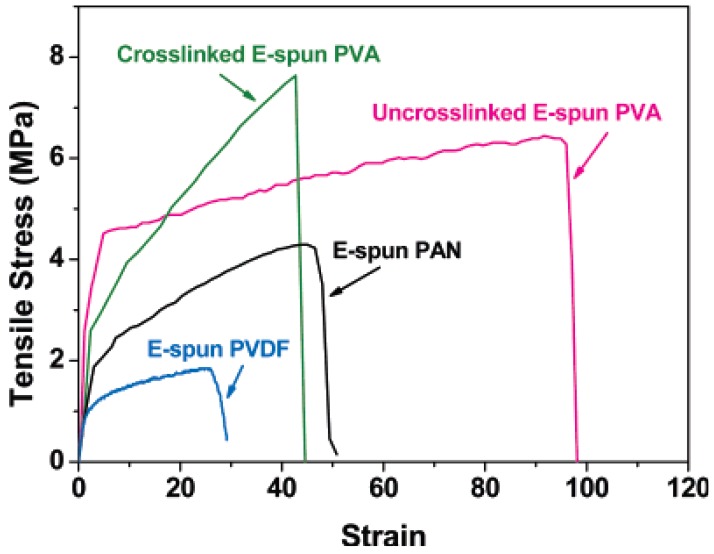
Tensile stress and strain curves of electrospun nanofibrous substrates (Adapted from [54]).

Recently, a novel three tier arrangement of composite membranes was developed. Water soluble polymer such as poly (vinyl alcohol) was electrospun on the non-woven microfibrous support and then chemically cross linked with glutraldehyde in acetone as a solvent. Due to its water soluble nature they were cross linked with glutraldehyde and resulted membranes exhibited excellent water resistance and mechanical properties [54]. The top layer of PVA membrane was coated with hydrophilic/MWNT and filtration test was conducted for selective water-oil emulsion separation. Figure 6 clearly explains that tensile strength of the with/without cross linked electrospun PVA was better than that of other nanofibers produced from polyvinylidenefluoride and polyacrylonitrile.

**Figure 7:**
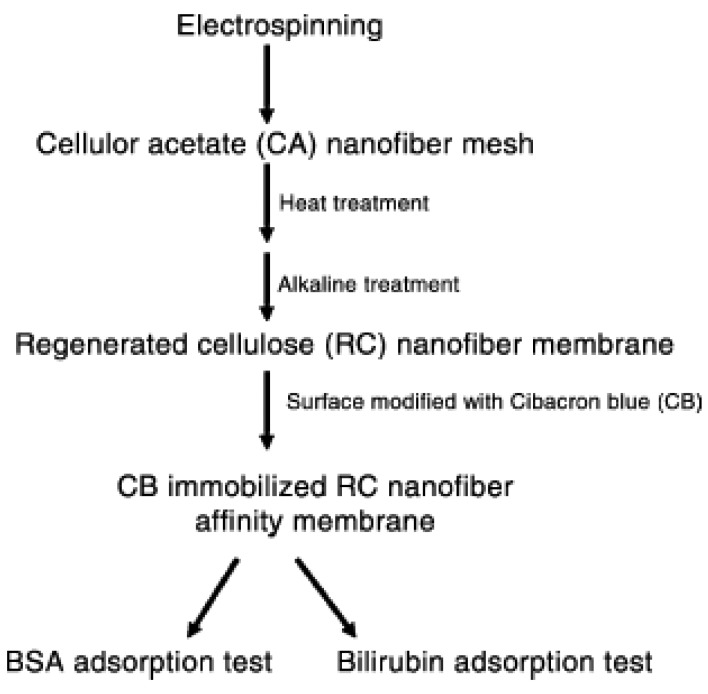
Schematic diagram of overall process of bio-removal by regenerated cellulose (Adapted from [13]).

As we mentioned earlier, electrospinning of natural polymer faces many obstacles due to their high molecular weight, high viscosity at low concentration and poor solubility in organic solvents. As with chitosan, natural polymer cellulose also faces similar problems like solubility as well as in electrospinning. Cellulose membranes are widely used in membrane preparation due to their hydrophilicity. To overcome this, cellulose was converted into acetylated derivative, commonly known as cellulose acetate. Ma *et al.* interestingly obtained regenerated cellulose from electrospun cellulose acetate further treating with alkaline solution. Before using these microfiltration membranes for removing biomolecules they were treated in high temperature followed by surface modification using Cibacron Blue F3GA dye which is used for removing bovine serum albumin and bilirubin [13]. The overall schematic processes involved in generating regenerated cellulose nanofibers are given in Figure 7.

**Figure 8:**
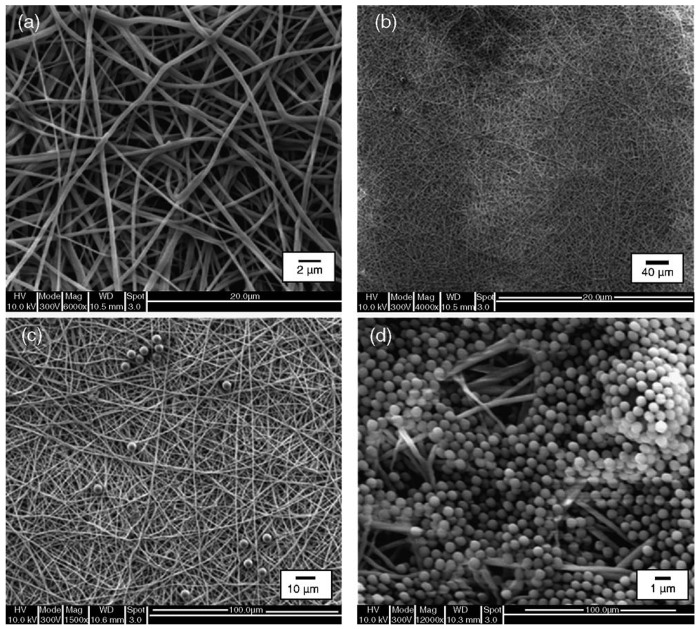
FESEM pictures of electrospun PVDF membrane **(a)** before separation, **(b)** after 10 μm, **(c)** after 5 μm, and **(d)** after 1 um polystyrene particle separation (Adapted from [8]).

Gopal *et al.* [8] have studied the electrospun polyvinylidene difluoride (PVDF) nanofibrous membranes for microfiltration of different micrometers size (10, 5, 1 μm) of polystyrene particles and PVDF surface morphologies before and after separations, as shown in Figure 8. The study also proves the efficiency of the electrospun nanofibers compared to the conventional microfiltration membranes with high rejection rate more than 90% of polystyrene micro particles. Overall studies and characterization explored the use of nanofibers as potential pre-treatment membranes in water separation technology.

Yoon *et al.* recently reported the electrospun polyacrylonitrile membrane with chitosan surface coating as novel high flux ultrafiltration membranes. Their studies clearly demonstrated that the electrospun UF/NF membranes with the top layer coated with hydrophilic water permeable chitosan, provide high flux. This method could replace conventional membranes due to their high flux in water filtration [55].

#### 2.1.8. Surface Modifications of Membranes

Generally, hydrophobic membranes can be modified to hydrophilic membranes by using various methods. Methodology used for conversion is known as hydrophilization. The method of hydrophilization is divided into four major types namely: (a) Plasma induced surface grafting (PISG) treatment; (b) Chemical oxidation; (c) Organic chemical surface functionalization; and (d) radiation induced surface grafting method [56,57,58,59]. Among these, plasma induced surface grafting by methacrylic acid was applied on the electrospun PVDF. Moreover, comparison studies made with commercially available hydrophobic PVDF membranes suggested that this technique can be used to reduce the pore size of the PVDF membranes. The studies have proven that by using plasma induced grafting technique, the pore size has been reduced when compared to commercial membranes due to their available surface volume ratio and also significant increase in flux [10]. Utilizing, the current state of the art nanofibrous membranes, for filtration areas and objectives for developing nanofiber based filters is extensively reviewed. They also addressed some of the problems in handling the nanofibrous membranes like pleating of the membranes, stability, longer efficiency, *etc.* [60]. Barhate *et al.* also discussed the various techniques involved in the preparation of surface modified electrospun nanofibrous membranes [61].

#### 2.1.9. Micro and Ultra Filtration: Particles and Heavy Metal Ions Separations

Recently, pre-filters gained more attention due to their high versatile applications over filtration of micro-particles from waste water. Polysulfone nanofibers were used as pre-filters prior to ultra/nano filtration for micro particle separation, which enhance the life of Ultra/Nano filtration membranes. Due to its high porosity with high surface area; they can be widely used as pre-filters [9]. Sang *et al.* recently reported the electrospun chloridized poly (vinyl chloride) nanofibers for copper (II) removal based on Micellar-enhanced filtration (MEF) with alumina adsorption. Utilizing the same nanofibers, they also discussed the micellar-enhanced filtration with 10 layer filtration and sodium dodecylbenzenesulfonate (SDBS) of 5 mmol/L for the removal of heavy metal ions such as copper, lead and cadmium [62,63]. The particle size plays an important role in determining the efficiency of the membranes and it is directly related to the flux and separation factor. A recent article explored the efficiency of the electrospun nylon-6 nanofibers as a pre-filter for performing the separation of particles ranging from 10 to 0.5 μm. They also discussed the pore size of the nanofibrous membranes and fouling resistance resulting from their separation [64].

On continuation of particle and heavy metal separation, Desai *et al.* reported the electrospinning difficulties of chitosan/poly(acrylamide) and overcame these by varying the parameters such as polymer concentration and temperature and thereby uniform beadless nanofibers were achieved. In an another work, potential use of electrospun chitosan/poly(ethylene oxide) (PEO) nanofibers for heavy metal ion binding, antimicrobial as well as physical separations were clearly examined. They also proved that filtration efficiency was strongly related to the size of the electrospun fibers and percentage of the chitosan present on the surface. They have utilized these membranes in binding hexavalent chromium ions [65,66,67]. Bjorge *et al.* evaluated electrospun nanofibrous membranes for water filtration applications. Their detailed study bridges the gap between the electrospinning techniques for preparation of flat sheet membranes and their application in water filtrations such as pathogen removal, suspended solids and as an alternative for conventional flat sheet membranes [68]. Among the researchers, the quest to develope nanofibrous membranes has been extensively increased and, as a result, various polymers have been electrospun and tested for their efficiencies by their application in different filtrations. Among them, polyethersulfone nanofibers have also been used for filtration studies with non-woven poly (ethylene terephthalate) as backing layer. As filtration is a pressure related process, the strength and stabilities have to be increased and this has been achieved by heat treatment. The heat treated membranes were characterized thoroughly and their efficiency was tested with polystyrene micro particles [69].

Ma *et al.* recently reported the ultra thin coating of low or poor soluble cellulose on the surface of PAN/PET using two ionic liquids namely, 1-butyl-3-methylimidazolium chloride and 1-ethyl-3-methylimidazolium acetate as solvents under extremely mild conditions. Further, the ultra thin cellulose coated ultrafiltration membranes were tested for separation of emulsified oil and water mixture and proved higher flux results with same rejection rates as compared with commercial UF membranes [70]. Using solvent vapor treatment method, hydrophilic PVA layer chemically cross linked with glutaraldehyde solution on a double layer of PVA/PAN nanofibrous composite was prepared and tested for oil/water systems and achieved 99.5% rejection with high permeate flux at 0.3 MPa operating pressure [71]. It is to be noted that all silver and its related ions are used as antimicrobial agents. Recently, Zhang *et al.* prepared nano silver coordinated amidoxime membranes by reacting hydroxylamine with nitrile groups present on the surface of PAN membranes and further modified with silver ions/nanoparticles. Results showed that the developed membrane have both antimicrobial activity as well as normal water transport properties [72].

#### 2.1.10. Future Directions and Conclusions

Currently, glass filters and activated charcoal are widely used in air filtration applications. The use of glass fibers still cause environmental and health problems [73,74] both in mechanical recycling and end-of-life disposal through incineration (thermal recycling). Hence environmentally friendly composite systems based on natural fibers can be used as an alternative to glass fibers. Also one of the limitations of HEPA filters, *i.e.* them not being applicable for the filtration of chemical contaminants, can be overcome by polymer based nanofibers embedded with nanoparticles. The charcoal based filter can be replaced with polymer based nanofibers embedded with nanoparticles. These polymer nanofibers embedded with nanoparticles will not only improve the filtration efficiency, but also the protection duration, nonselective decontamination efficiency, and weight reduction. Sensor embedded protective clothing should be developed which would provide both protection and sensing. Development of the methodologies and technologies, in such a way that terrorists are unable to access them, is currently one of the foremost requirements. For instance, for protection against genetically modified biological contaminants potentially deployed on soldiers or the public. The development of catalysts to decontaminate both chemical and biological warfare agent's area for air filter applications should be focused in future both by academic labs and industries. In the water research area, one of the major problems faced by this challenging world is availability of drinking water and several methods of purification technologies have emerged.

The thin film nanocomposite membranes (TFNC) developed in our laboratory showed higher water flux than the commercial NF270 and NF90 membranes with slightly lower rejection. Recently, we have shown that by decreasing the thickness of the nanofibers and fiber diameter, improved flux can be achieved for TFNC membranes. The next generation membranes for water treatment applications would be based on nanofibrous membranes (especially TFNC), which would be cost effective and energy saving to use prior to reverse osmosis membranes. Recent advancements in nanofibrous membrane preparation, paved the way for a large number of water filtration systems producing safe and clean water. Apart from normal filtration systems they are exploited in several bio-removal processes, as well as in environmental waste water purification systems; they are also used in waste water treatment applications. Due to their ease of operation and greater efficiency, they will play an important role in the replacement of conventional membranes in the near future.
